# Identification and characterization of the structure genes involved in anthocyanin biosynthesis in flowers of *Cymbidium* species

**DOI:** 10.3389/fpls.2026.1776466

**Published:** 2026-02-13

**Authors:** Kunkun Zhao, Junyi Xie, Yihong Zheng, Xiaodong Yang, Yunzhu Wang, Xue Zhang

**Affiliations:** 1 College of Architecture, Anhui Science and Technology University, Chuzhou, China; 2 College of Environmental Ecology, Jiangsu Open University, Nanjing, China; 3 Zhejiang Academy of Agricultural Sciences, State Key Laboratory for Quality and Safety of Agro-Products, Hangzhou, China; 4 MOE Key Laboratory of Freshwater Fish Reproduction and Development, School of Life Sciences, Southwest University, Chongqing, China

**Keywords:** 2-ODD gene family, anthocyanin biosynthesis, Cymbidium, flower color, structural genes

## Abstract

The Orchidaceae family is renowned for its remarkable floral diversity, showing a wide range of colors primarily influenced by pigments especially anthocyanins. *Cymbidium haematodes* is a terrestrial orchid characterized by vibrant flower colors, making it an ideal resource for studying anthocyanin biosynthesis in orchids. In this study, we systematically identified and characterized the structural genes involved in anthocyanin biosynthesis in *C. haematodes* and compared their roles between *C. haematodes* and its close relative *C. sinense*. Pigment analysis revealed that anthocyanins were the predominant pigments in the red-colored tepals of *C. haematodes*, exhibiting significantly higher levels in dark red (DR) and red (R) accessions compared to lighter color accessions. Transcriptome sequencing identified 20 structural genes, including *CHS*, *F3H*, *F3’H*, *DFR*, *ANS*, and *UFGT*. Phylogenetic analysis indicated close evolutionary relationships with other *Cymbidium* species. Notably, *CsF3’H2* and *CsF3’H3* exhibited high expression levels in red tepals, while *CsUFGT1–3* and *CsDFR1–2* were up-regulated in *C. sinense*. This suggests species-specific regulatory mechanisms governing pigment production. Subcellular localization assays confirmed cytoplasmic distribution for *CsANS*, chloroplast localization for *CsF3’H2*, and cell membrane association for *CsF3’H3*, implicating diverse functional roles related to anthocyanin transportation and biosynthesis. These findings highlight the functional divergence of structural genes in anthocyanin biosynthesis between *C. haematodes* and *C. sinense*, and have specifically identified *CsF3’Hs* as key genes of red coloration in *C. haematodes* flowers. This study provides foundational insights into the molecular mechanisms underlying flower color variation among orchids, offering potential targets for future genetic manipulation aimed at enhancing ornamental breeding practices.

## 1 Introduction

Orchidaceae is recognized as one of the three largest families in angiosperms, compromising 899 genera and 27,801 species, which collectively account for approximately 10% of flowering plant (Phillips et al., 2020; Zhao et al., 2023). The members of the Orchidaceae family, commonly referred to as orchids, have become globally renowned and widely cultivated due to their exquisitely unique floral structures, remarkable diversity in colors variations, and distinctive fragrances. Flower color represents one of the most significant ornamental traits in orchids, and is typically determined by a combination of internal factors, such as epidermal cell morphology, intracellular structures, pigment composition, and external environmental factors (Zhou et al., 2021). Among these determinants, the types and concentrations of floral pigments directly influence the manifestation of flower color. In orchids, the primary pigments responsible for coloration include carotenoids, chlorophylls, and flavonoids (Hsiao et al., 2011). Flavonoids, in particular, are a group of compounds characterized by a core structure known as 2-phenylchromone, and encompass various subclasses such as anthocyanins, flavones, and flavonols.

Anthocyanins are natural water-soluble pigments that exhibit a wide spectrum of colors ranging from red, orange, purple to blue. These pigments typically exist in the form of glycosides within the epidermal cells of plants. To date, over 600 distinct types of anthocyanins have been identified and characterized (Zhou et al., 2021). Among them, the three most prevalent anthocyanins are delphinidin-3-glycoside (blue/violet), cyanidin-3-glucoside (brick red/magenta) and pelargonidin-3-glycoside (orange/red). Additionally, petunidin, malyidin and peonidin are derived from these primary compounds. Anthocyanins are widely distributed across more than 70 taxa and 27 families of plant species, found in leaves, stems, roots, flowers, and fruits (Zuo et al., 2012). As one of the most extensively distributed secondary metabolites in land plants, anthocyanins play a crucial role in plant color determination, pollinator attraction and the resistance of abiotic and biotic stresses (Li et al., 2022). Furthermore, anthocyanins possess health-promoting properties due to their antioxidant capabilities (Pojer et al., 2013; Khoo et al., 2017). Recent studies have focused on flower color-related research in Orchidaceae species, such as *Cymbidium ensifolium* (Ai et al., 2023), *Phalaenopsis Aphrodite* (Wang et al., 2018; Hsu et al., 2019), and *Dendrobium officinale* (Li et al., 2023; Yang et al., 2023), suggesting that peonidin, pelargonidin, and cyanidin are the primary floral pigments in orchids.

The anthocyanin biosynthesis pathway (ABP) is an important branch of the phenylpropanoid pathway and is genetically controlled by two types of genes: structural genes and regulatory genes. The ABP typically comprises three stages, including phenylalanine and phenylpropanoid metabolism, flavonoid metabolism, and anthocyanin metabolism (Grotewold, 2006). The first stage starts with the general phenylpropanoid pathway, which is catalyzed by phenylalanine ammonia lyase (PAL) and cinnamate-4-hydroxylase (C4H), leading to the production of 4-coumaroyl-CoA. In the second stage, 4-coumaroyl-CoA serves as the substrate for chalcone synthase (CHS) and chalcone isomerase (CHI). The product is naringenin which is a general precursor of the phenylpropanoid pathway branches such as flavones, isoflavones, flavonols, and anthocyanins (Davies et al., 2020). Naringenin is then converted into dihydrokaempferol (DHK) through the action of flavanone 3-hydroxylase (F3H). Flavonoid 3*’*-hydroxylase (F3*’*H) and flavonoid 3*’*, 5*’*hydroxylase (F3*’*5*’*H) further catalyze DHK into dihydroquercetin (DHQ) and dihydromyricetin (DHM), respectively. The final stage involves catalysis by dihydroflavonol reductase (DFR) and anthocyanidin synthase (ANS), yielding three types of products: delphinidins, pelargonidins, and cyanidins. Among genes encoding these enzymes, the early biosynthetic genes (EBGs) include *CHS*, *CHI*, *F3H* and *F3’H*, which are regulated by the MYB transcription factors in plants (Yan et al., 2021). Conversely, the late biosynthesis genes (LBGs) such as *DFR*, *ANS*, and *UFGT* are modulated by the MYB-bHLH-WD40 (MBW) complex which influences downstream anthocyanin accumulation in plants (Petroni and Tonelli, 2011; Shin et al., 2013; Hui et al., 2019). These enzymes belong to different functional classes. For example, CHS belongs to polyketide synthases, while DFR is short-chain dehydrogenases/reductases. Additionally, F3H, ANS, and FLS are all categorized as 2-oxoglutarate-dependent dioxygenases (2-ODD). Both F3*’*H and F3*’*5*’*H are members of the cytochrome P450 monooxygenases family, classified into the CYP75B and CYP75A subfamilies, respectively (Winkel, 2006). In orchids, several structural genes have been identified and confirmed to be associated with anthocyanin biosynthesis in some species, such as *Oncidium* (*OgCHS*) (Liu et al., 2012), *Paphiopedilum concolor* (*PcCHS*) (Li et al., 2012), *Vanda hybrid* (*Va-F3H1*) (Junka et al., 2011), *P. Aphrodite* (*PhF3’5’H*, *PhF3’H* and *PeUFGT3*) (Chen et al., 2011; Yang et al., 2013). However, the understanding of flower coloration in *Cymbidium* species remains quite limited.

*C. haematodes* is a terrestrial perennial orchid belonging to the genus *Cymbidium*, characterized by its remarkably diverse flower colors, which range from red and pink to yellow, green, and white, all of which emit a strong fragrance. The diverse floral coloration and attractive fragrance of *C. haematodes* make it an excellent ornamental orchid with significant horticultural and economic values. The taxonomic classification of *C. haematodes* has been a subject of debate over time. It was initially classified as a subspecies of *C. ensifolium* (*C. ensifolium* subsp. *haematodes*) and later regarded as a variety of *C. sinense* (*C. sinense* var. *haematodes*). However, *C. haematodes* is now widely recognized as a distinct species (Wu and Raven, 2013), and is closely related to *C. sinense*. In this study, we identified and characterized 20 structural genes involved in the anthocyanin biosynthesis pathway in *C. haematodes* through transcriptome sequencing, bioinformatic analysis and quantitative real time PCR (qRT-PCR) analysis. Our findings elucidate the divergent functional roles of these structural genes in the anthocyanin biosynthesis pathway of the genus *Cymbidium*, as well as providing insights into the mechanisms underlying flower coloration in Orchidaceae species.

## 2 Results

### 2.1 Analysis of pigment contents in tepals of Cymbidium species

**Figure 1:**
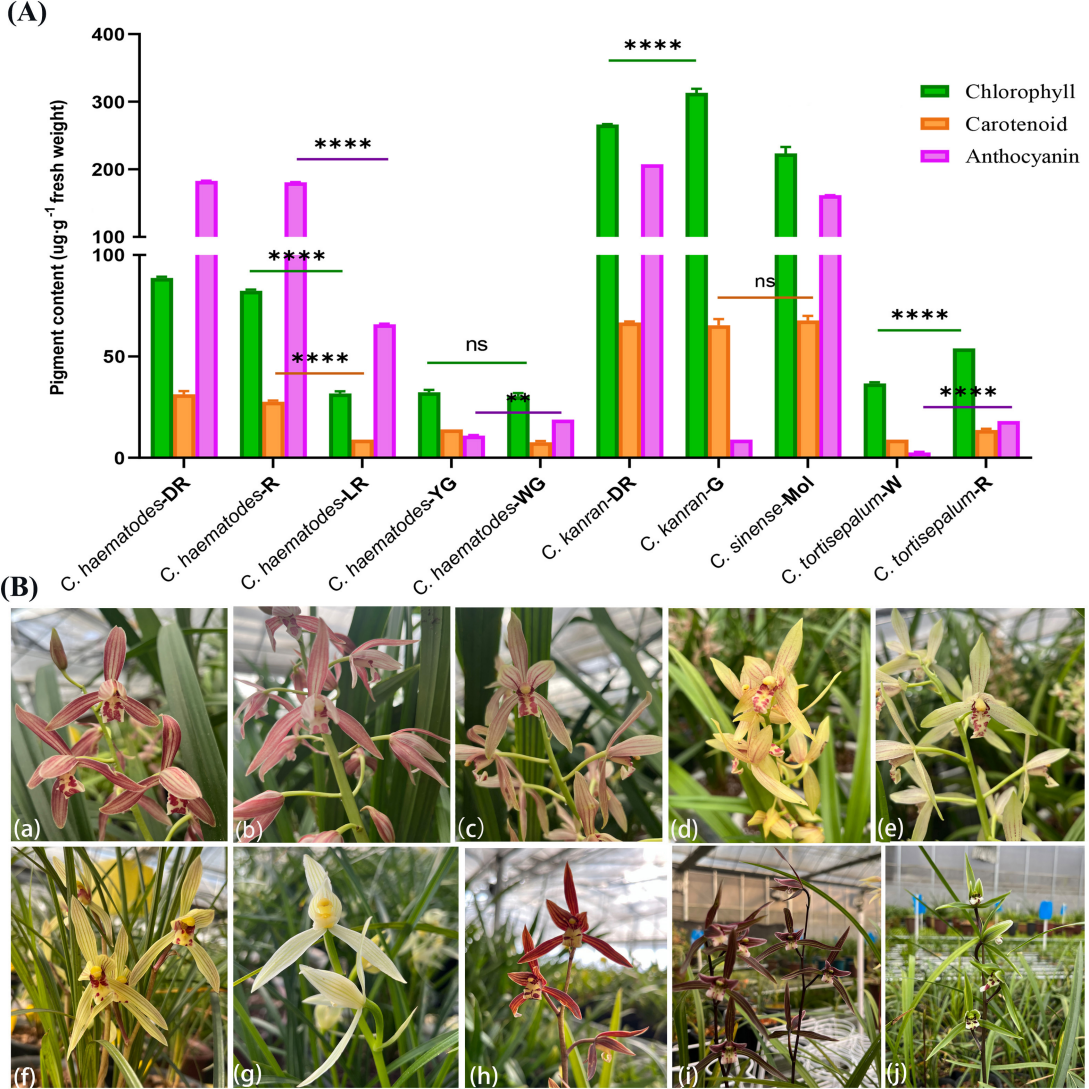
Pigment composition and content in tepals of *Cymbidium* species with different colors. **(A)** pigment content including chlorophyll (green), carotenoid (orange), and anthocyanin (purple); **(B)***Cymbidium* species for pigment detection, *C*. *haematodes*-DR (a), *C*. *haematodes*-R (b), *C*. *haematodes*-LR (c), *C*. *haematodes*-YG (d), *C*. *haematodes*-WG (e), *C*. *tortisepalum*-R (f), *C*. *tortisepalum*-W (g), *C. sinense*-Mol (h),*C. kanran*-DR (i), and *C. kanran*-G (j). * indicates *p* < 0.05, ** indicates *p* < 0.01, *** indicates *p* < 0.001, and **** indicates *p* < 0.0001.

To elucidate the composition of pigments in the floral tepals (petals and sepals), the contents of anthocyanins, carotenoids, and chlorophylls were measured in different *Cymbidium* species with various flower colors, including *C. haematodes*, *C. kanran*, *C. tortisepalum*, and *C. sinense* (**Table 1** and **Figure 1**).

**Table 1:** Pigment composition and content in tepals of different colors of *Cymbidium* species.

Number	Sample	Chlorophyll content (μg ·g^-1^ FW)	Mean ± SD	Carotenoid content (μg ·g^-1^ FW)	Mean ± SD	Anthocyanin content (μg·g^-1^ FW)	Mean± SD
1	*C. haematodes*-DR	89.42	88.80 ± 0.59	32.87	31.30 ± 1.56	182.65	182.94 ± 0.31
2		88.25		31.27		183.25	
3		88.72		29.75		182.93	
4	*C. haematodes*-R	81.91	82.31 ± 0.71	27.68	27.54 ± 0.24	180.83	180.84 ± 0.20
5		81.91		27.68		181.03	
6		83.13		27.27		180.64	
7	*C. haematodes*-LR	30.56	31.35 ± 1.36	8.78	8.78 ± 0.01	66.10	65.83 ± 0.28
8		30.56		8.78		65.84	
9		32.92		8.77		65.54	
10	*C. haematodes*-YG	33.17	32.51 ± 1.14	13.68	13.80 ± 0.20	11.21	10.91 ± 0.29
11		31.19		14.02		10.90	
12		33.17		13.68		10.63	
13	*C. haematodes*-WG	29.90	30.29 ± 1.32	8.35	7.70 ± 0.61	18.87	18.87 ± 0.00
14		29.21		7.59		18.87	
15		31.77		7.15		18.87	
16	*C. kanran*-DR	267.20	266.33 ± 0.75	66.11	66.57 ± 0.42	207.48	207.47 ± 0.01
17		265.90		66.65		207.48	
18		265.90		66.95		207.45	
19	*C. kanran*-G	319.80	313.40 ± 5.6	61.91	65.08 ± 2.89	8.95	8.97 ± 0.03
20		309.40		65.77		8.95	
21		311.01		67.56		9.00	
22	*C. sinense*-Mol	213.21	223.75 ± 9.15	64.65	67.52 ± 2.49	161.84	161.84 ± 0.14
23		228.41		69.06		161.98	
24		229.64		68.85		161.70	
25	*C. tortisepalum*-W	36.82	36.71 ± 0.19	9.18	9.13 ± 0.09	2.94	2.63 ± 0.27
26		36.50		9.02		2.42	
27		36.82		9.18		2.55	
28	*C. tortisepalum*-R	54.38	54.32 ± 0.10	13.54	13.54 ± 0.09	18.13	18.13 ± 0.00
29		54.21		13.46		18.13	
30		54.38		13.64		18.13	

The results indicated that *C. kanran*-G exhibited the highest chlorophyll content (313.40 μg/g·FW), while the white-green (WG) of *C. haematodes* showed the lowest chlorophyll content of 30.29 μg/g**·**FW (**Table 1**). The highest carotenoid content was 67.52μg/g observed in *C. sinense*, while *C. kanran*-DR and *C. kanran*-G exhibited comparable levels. On the other hand, the lowest carotenoid content was found in *C. haematodes*-WG at7.70 μg/g·FW. Anthocyanins are critical coloration compounds within flavonoids that play important roles in the color formation as well as various biological processes. Notably high levels of anthocyanins were detected in *C. kanran*-DR, *C. sinense*, and *C. haematodes*-R, with the highest anthocyanin content of 207.47 µg/g·FW found in the tepals of *C. kanran*-DR. In contrast, *C. tortisepalum*-W exhibited the lowest anthocyanin content (2.63 µg/g·FW).

The results showed that the anthocyanin contents are significantly higher in red tepals of *C. haematodes* (DR, R, and LR) than those of YG and WG. The chlorophyll and carotenoid contents in *C. haematodes* are relatively low, with no significant difference among different colors. This indicated that chlorophyll and carotenoid have minimal influence on the flower color variations within *C. haematodes*. The anthocyanin contents in *C. haematodes* exhibit considerable variation across different colors, being highest in DR, followed by R and LR, while YG and WG display the lowest levels. The petals of *C. sinense* ‘Qihei’ is claret colored, which is darker than that of DR. However, it was found that anthocyanin content of DR reached 182.94 μg·g^-1^·FW, whereas that in ‘Qihei’ was 161.84 μg·g^-1^**·**FW. Thus, there might be other pigments except for anthocyanins, carotenoids and chlorophylls that determine the flower color differences between *C. sinense* and *C. haematodes*. These findings suggest that the anthocyanin concentrations are the main factors that contributed to different flower colors of *C. haematodes*. The wide spectrum of flower color ranges in *C. haematodes* makes it a good resource for studying flower color formation within *Cymbidium* species. Thereby, we performed further analysis to explore the mechanisms regulating anthocyanin biosynthesis in *C. haematodes* flowers.

### 2.2 Identification of key structural genes for anthocyanin biosynthesis in *C. haematodes*

Because no reference genome has been published for *C. haematodes*, and given its close relationship to *C. sinens*e, we conducted transcriptome sequencing of the *C. haematodes* tepals using *C. sinens*e genome as the reference genome. We also performed sequence comparisons between *C. haematodes* and *C. sinens*e. Transcriptome sequencing was carried out on 15 samples representing five different color accessions of *C. haematodes* (DR, R, LR, YG, and WG). The comparison rates for 15 samples against the reference genome of *C. sinens*e all exceeded 85% (**Table 2**), indicating that the genome met the requirements for gene analysis related to anthocyanin biosynthesis in *C. haematodes*.

**Table 2:** The mapping rates of each sample in the transcriptome sequencing with reference genome.

Sample	Total reads	Reads mapped	Mapping rate
DR-1	54,031,780	46,859,587	86.73%
DR-2	74,874,760	64,791,508	86.53%
DR-3	75,529,288	65,356,404	86.53%
LR-1	55,308,868	47,450,980	85.79%
LR-2	69,294,198	59,404,001	85.73%
LR-3	56,185,356	48,197,101	85.78%
R-1	80,592,528	69,444,176	86.17%
R-2	57,339,252	49,645,440	86.58%
R-3	61,095,028	53,278,335	87.21%
WG-1	115,999,374	100,613,761	86.74%
WG-2	100,710,616	87,724,095	87.11%
WG-3	66,334,508	57,715,721	87.01%
YG-1	74,224,520	63,988,288	86.21%
YG-2	82,005,190	70,926,219	86.49%
YG-3	80,209,858	69,296,124	86.39%

**Figure 2:**
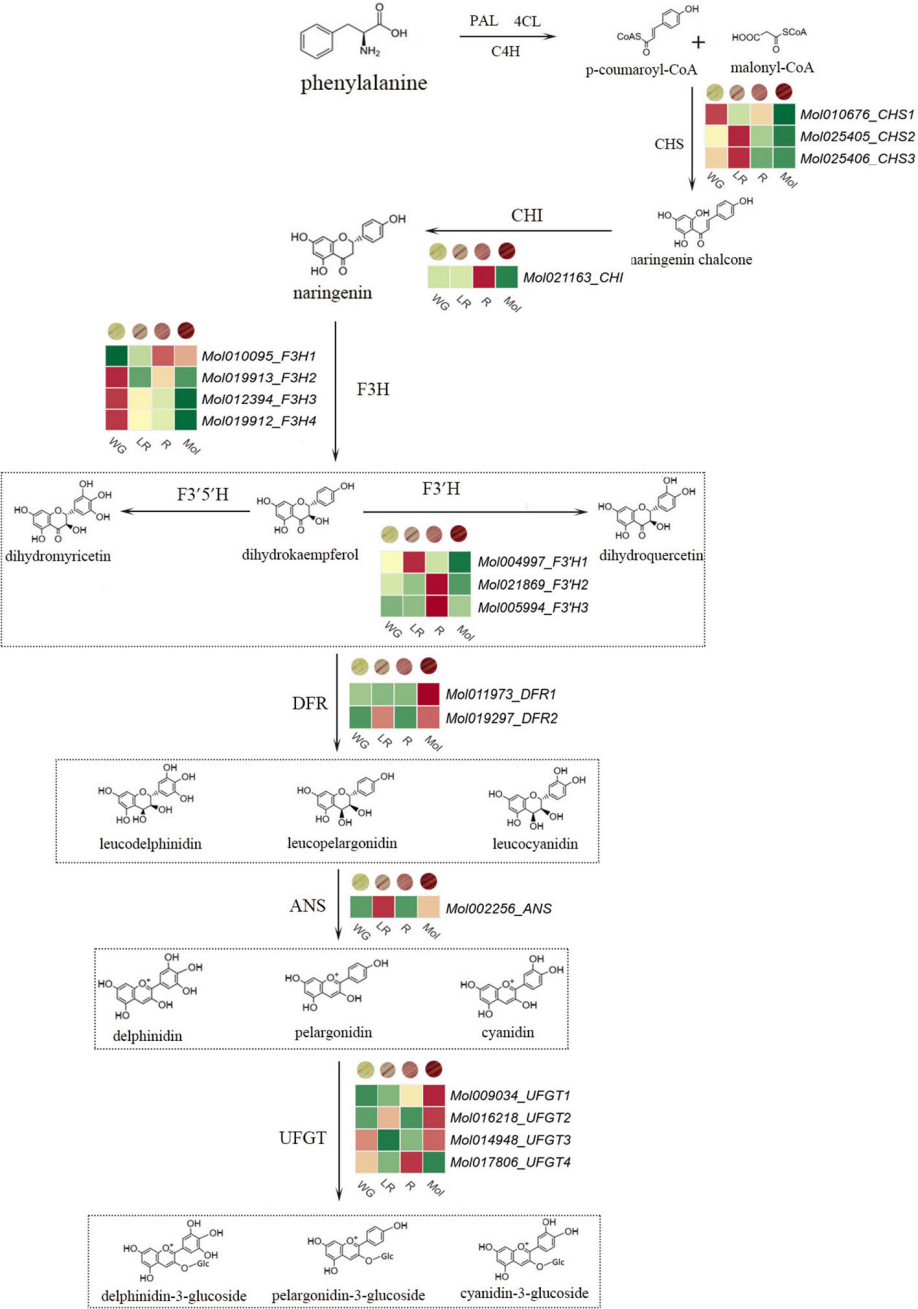
Anthocyanin biosynthesis pathway.

According to the annotation information from the NR database of *C. haematodes*, a total of 20 structural genes related to anthocyanin biosynthesis pathway were identified (**Figure 2**). This includes three *CsCHS* genes, one *CsCHI* gene, four *CsF3H* genes, two *CsFLS* genes, three *CsF3’H* genes, two *CsDFR* genes, one *CsANS* gene and four *CsUFGT* genes (**Table 3**). These genes encode flavonoid biosynthesis enzymes belonging to different functional classes. For example, the CsCHS proteins belong to polyketide synthases, while CsDFR proteins are short-chain dehydrogenases/reductases. Additionally, CsF3H, CsANS and CsFLS proteins belong to 2-ODDs. The results of physical and chemical property analysis showed that nearly half of the proteins encoded by structural genes were unstable proteins with instability coefficient greater than 40. Moreover, most proteins were hydrophilic proteins with average hydrophilic index less than 0. The longest proteins were the three F3*’*Hs, with 515–531 aa in length. Subcellular localization of the proteins encoded by structural genes was predicted on cytoplasm, nucleus and chloroplast, among which over 65% proteins located on cytoplasm. Only two F3H proteins were predicted on nucleus, whereas all the F3*’*H proteins, one DFR, and one UFGT were located on chloroplast.

**Table 3:** Molecular characterization of the structural genes related to anthocyanin biosynthesis.

Gene ID	Gene	Amino acid aa	Molecular weight	pl	Instability index	GRAVY	Subcellular location
Mol010676	*CsCHS1*	262	28506.87	8.54	42.42	-0.129	cytoplasm
Mol025405	*CsCHS2*	251	27172.02	5.62	58.56	-0.098	cytoplasm
Mol025406	*CsCHS3*	93	10931.63	8.71	62.01	-0.400	cytoplasm
Mol021163	*CsCHI*	185	19709.45	4.87	45.40	-0.109	cytoplasm
Mol010095	*CsF3H1*	222	25069.36	5.98	57.48	-0.359	nucleus
Mol019913	*CsF3H2*	442	49089.57	5.66	38.87	0.078	cytoplasm
Mol012394	*CsF3H3*	350	39691.17	5.17	54.36	-0.409	nucleus
Mol019912	*CsF3H4*	270	30689.89	4.85	49.89	-0.171	cytoplasm
Mol004997	*CsF3’H1*	530	58296.47	9.24	35.56	-0.070	chloroplast
Mol021869	*CsF3’H2*	515	57138.31	6.75	43.98	-0.036	chloroplast
Mol005994	*CsF3’H3*	531	58947.94	8.47	38.51	-0.014	Cytoplasm
Mol002504	*CsFLS1*	333	37697.4	5.81	43.6	-0.35	cytoplasm
Mol011301	*CsFLS2*	150	17464.95	7.85	33.53	-0.541	cytoplasm
Mol011973	*CsDFR1*	276	29758.38	7.63	31.08	0.076	chloroplast
Mol019297	*CsDFR2*	332	37770.21	5.30	38.04	-0.240	cytoplasm
Mol002256	*CsANS*	359	39782.36	5.41	37.47	-0.218	cytoplasm
Mol009034	*CsUFGT1*	422	46355.66	5.70	38.55	0.175	chloroplast
Mol016218	*CsUFGT2*	452	49522.71	6.88	53.60	0.062	cytoplasm
Mol014948	*CsUFGT3*	230	26412.11	6.55	39.52	-0.461	cytoplasm
Mol017806	*CsUFGT4*	479	53526.99	5.90	48.27	-0.115	cytoplasm

### 2.3 Phylogenetic analysis of key structural genes for anthocyanin biosynthesis

**Figure 3:**
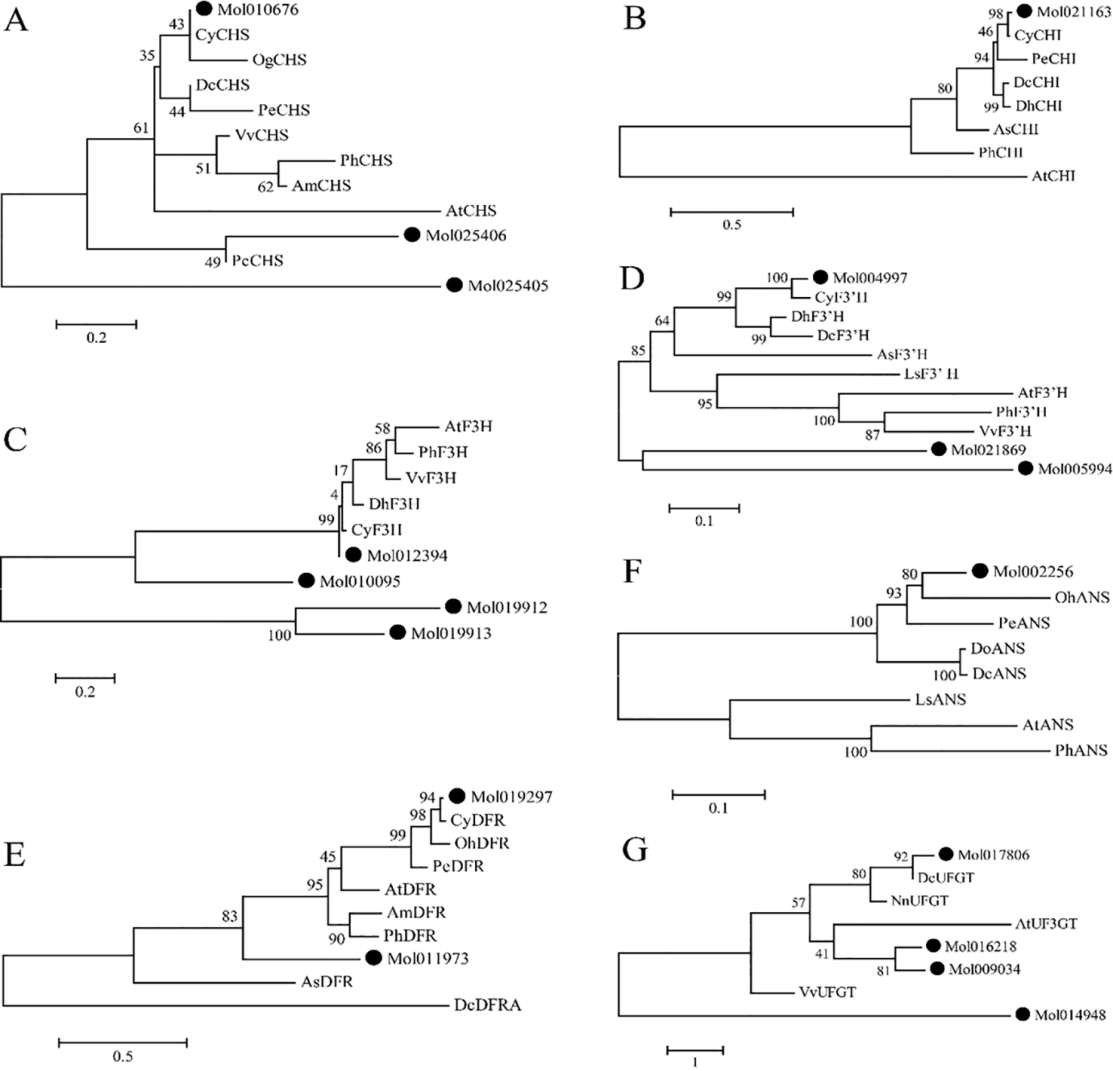
Phylogenetic analysis of proteins of seven structural genes CsCHS **(A)**, CsCHI **(B)**, CsF3H **(C)**, CsF3’H **(D)**, CsDFR **(E)**, CsANS **(F)**, and CsUFGT **(G)** in *C*. *haematodes* and those in other species.

To investigate the evolution relationship of the structural genes in anthocyanin biosynthesis pathway, phylogenetic analysis was performed for amino acid sequences of the seven types of structural genes encoded proteins, including CHS, CHI, F3H, F3’H, DFR, ANS, and UFGT. A total of 66 protein sequences from 13 plant species were used to construct seven phylogenetic trees (one tree for each type of structural genes). In general, at least one structural gene from *C. haematodes* is most closely clustered with *C. hybrid* which is also from genus *Cymbidium* (**Figure 3**). For example, the CHS protein sequences from 10 species were used to construct a phylogenetic tree. The result shows that the CsCHS1 is closely related to CyCHS from *C. hybrid*, indicating high homology within the same genus. Both of them are clustered together with *Oncidium* (**Figure 3A**). However, the other two CsCHS proteins are distance from most Orchidaceae species. Similar patterns are also observed in the phylogenetic trees of CHI, F3H, F3H and DFR (**Figures 3C–E**). Nonetheless, CsANS was clustered together with *Oncidium* OhANS (**Figure 3F**), and CsUFGT4 showed high homology with *Dendrobium* (**Figure 3G**).

**Figure 4:**
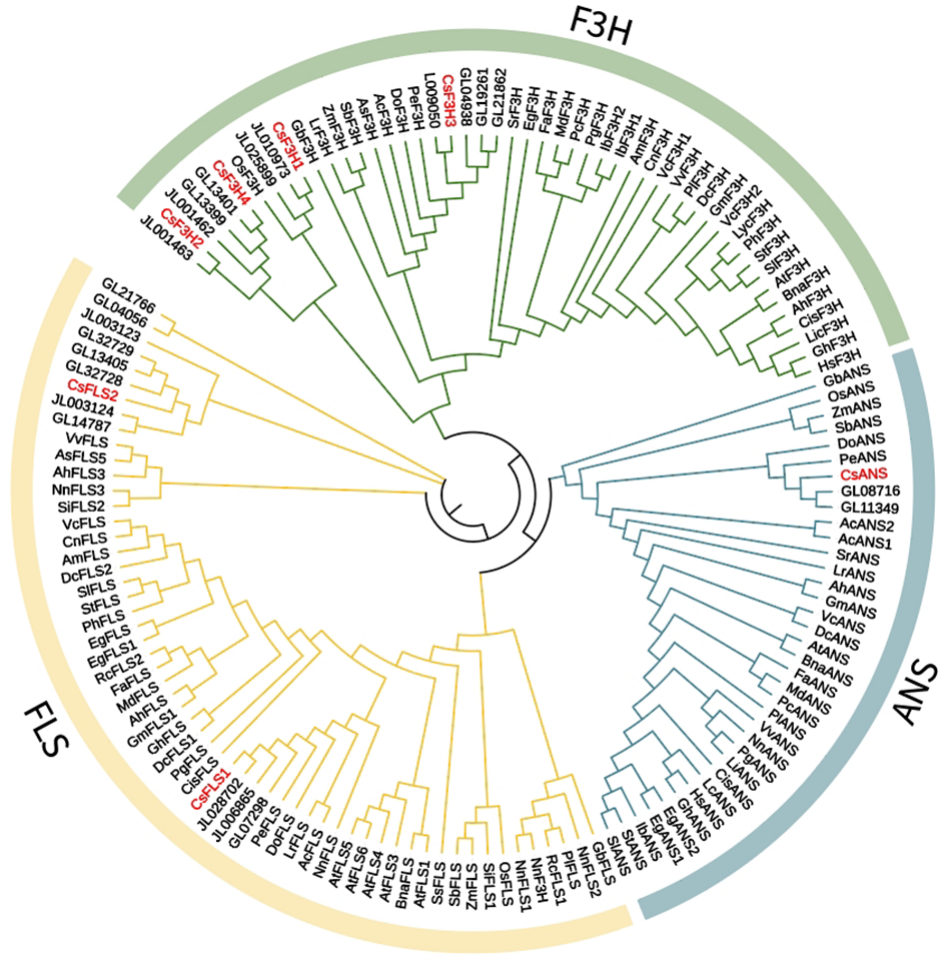
Phylogenetic analysis of the 2-ODD family members in 43 plant species. The phylogenetic tree was constructed using MEGA X with the maximum-likelihood (ML) method and 1000 bootstrap replicates. The 2-ODD proteins were grouped into FLS, F3H and ANS. The families are indicated by different arc line and branch colors.

Moreover, a phylogenetic tree was also constructed to analyze the evolutionary relationships between *C. haematodes* 2-ODDs and 142 flavonoid biosynthesis-related 2-ODDs including monocotyledonous and dicotyledonous species (**Figure 4**). The proteins are mainly grouped into two major clades, corresponding to the DOXC28 and DOXC47 subgroups of the 2-ODD superfamily. In the F3H-containing DOXC28 group, the CsF3Hs are clustered with F3H proteins from Orchidaceae species *Cymbidium*, and those from other monocots. Similar patterns are also observed for the CsANSs and CsFLSs. Among the orchids, the *C. haematodes* 2-ODDs are most closely related to the 2-ODDs from *Cymbidium* species, indicating high homology and possible similar functional roles.

### 2.4 Conserved motif and gene structure analysis of the 2-ODD proteins

**Figure 5:**
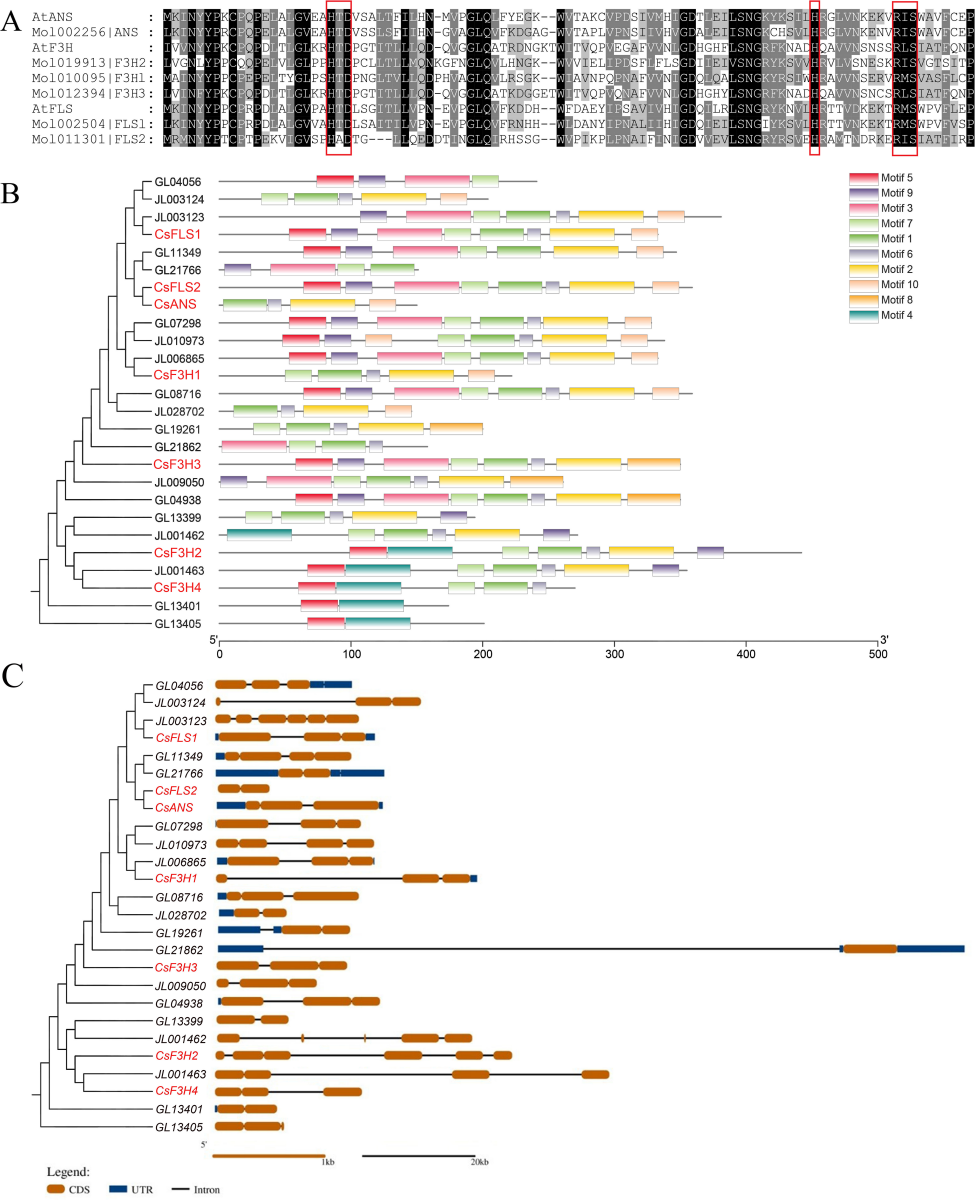
Sequence alignment **(A)** and analysis of the conserved motifs **(B)** and gene structure **(C)** of the *2-ODDs* in three *Cymbidium* species including *C*. *haematodes*, *C*. *goeringii*, and *C*. *ensifolium*.

Multiple sequence comparison results showed that all six 2-ODD proteins contained H-x-D-xn-H and R-x-S conserved residues, which are the binding sites for Fe^2+^ ions and 2-oxoglutarate, respectively (**Figure 5A**). These two conserved residues are well known for the highly conserved sequences among most plant 2-ODD proteins. Conserved motif analysis of the 2-ODD proteins of three *Cymbidium* species, *C. haematodes*, *C. goeringii*, and *C. ensifolium* showed that most 2-ODD protein sequences had four motifs: motif1, motif2, motif6, and motif7, suggesting that these motifs might be the most conserved amino acid residues of the *Cymbidium* 2-ODDs (**Figure 5B**). All terminals of CsFLS, CsANS and CsF3H1 contain motif10, while the end motif of CsF3H3 is motif8, which might be a specific motif. Meanwhile, two F3H proteins (Mol019913/CsF3H2, Mol019912/CsF3H4) contain a specific motif (motif4). Among them, Mol019912 that located in the same evolutionary branch may have lost some sequences due to evolution or assemble mistakes. The above results indicate that there are diversity and differences in the sequences of the 2-ODD family protein among different species, which closely related to the functional diversity of those genes.

Analysis of the exon/intron structural patterns in homologous genes of three *Cymbidium* species (**Figure 5C**) revealed that the number of exons ranged from 2 to 6, suggesting exon loss or gain during gene evolution. The most common exon numbers were two or three, with 14 genes (53.8%) exhibiting 3 exons and 2 introns. Notably, *JL001462* displayed a 5-exon and 4-intron structure, while *CsF3H2* and *JL003123* contained the highest number of exons and introns, with 6 exons and 5 introns. Furthermore, each gene possessed varying lengths of coding sequences (CDS). In contrast, untranslated regions (UTRs) were either short or entirely absent, with lower sequence similarity compared to CDS. This indicates that coding regions exhibit higher evolutionary conservation than UTRs.

### 2.5 Analysis of expression patterns of structural genes in anthocyanin biosynthesis

**Figure 6:**
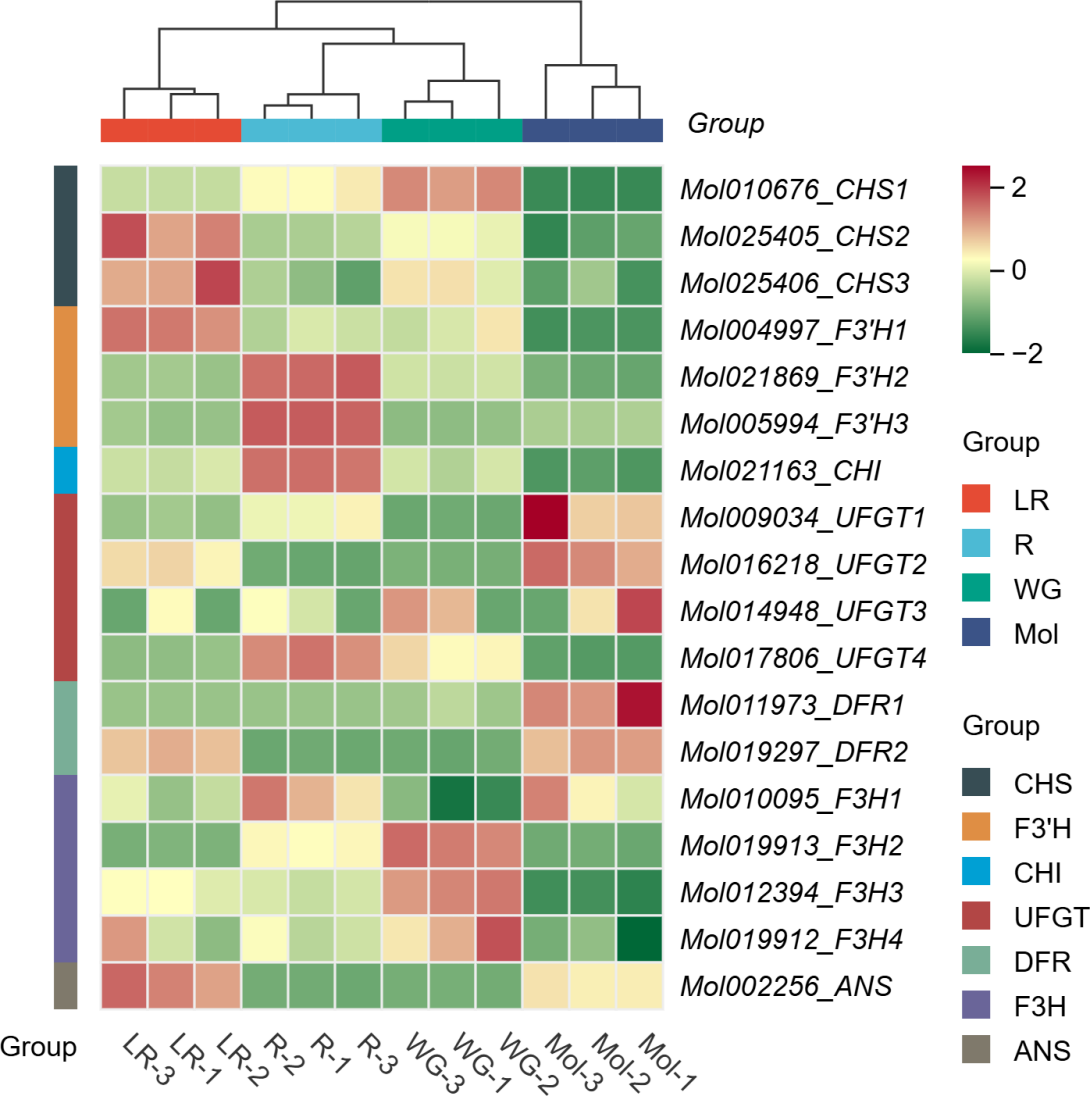
Gene expression profiles of 18 structural genes in tepals of different flower colors of *C. haematodes* (R, WG, and LR) and *C. sinense* (Mol).

To understand the potential roles of the structural genes involved in regulating anthocyanin biosynthesis, expression profiles of the structural genes in anthocyanin biosynthesis pathway were analyzed by using transcriptome sequencing data. Transcriptome sequencing was performed by using the tepals of three *C. haematodes* accessions with different colors (R, LR, and WG) and *C. sinense* ‘Qihei’. The results revealed distinct expression patterns of the structural genes in different colors of *C. haematodes* and *C. sinense* tepals (**Figure 6**). Four genes showed similar expression patterns. For example, *CsF3’H3* and *CsF3’H2* exhibit exclusive high expression levels in R tepals, whereas minimal expressions in other samples. *CsUFGT4* and *CsCHI* also had relatively high expression levels in R, compared to LR, WG and *C. sinense*. Interestingly, the rest three *CsUFGTs* all highly expressed in the tepals of *C. sinense*, indicating functional divergence in the same gene subgroup. Except for the three *CsUFGTs*, *CsDFR1* and *CsDFR2* also had high expression levels in *C. sinense*, indicating there might be specific roles of these LBGs in anthocyanin biosynthesis in *C. sinense*. Moreover, *CsDFR2* and *CsUFGT2* also showed moderate to low expression in LR. Among three *CsCHS* genes, high expression levels of *CsCHS2* and *CsCHS3* in LR were detected, but *CsCHS1* was slightly expressed in WG. These findings suggest that the structural genes have experienced functional divergence in regulating anthocyanin biosynthesis within the same subgroups and among different colors of *C. haematodes* and *C. sinense*. Despite that *C. haematodes* and *C. sinense* are closely related species, the molecular mechanisms in regulating flower color are obviously different. *CsF3’H3* and *CsF3’H2* are suggested as the potential key genes for anthocyanin biosynthesis of *C. haematodes*. However, further gene function verifications remain to be conducted.

### 2.6 qRT-PCR analysis of the structural genes in *C. haematodes* tepals

**Figure 7:**
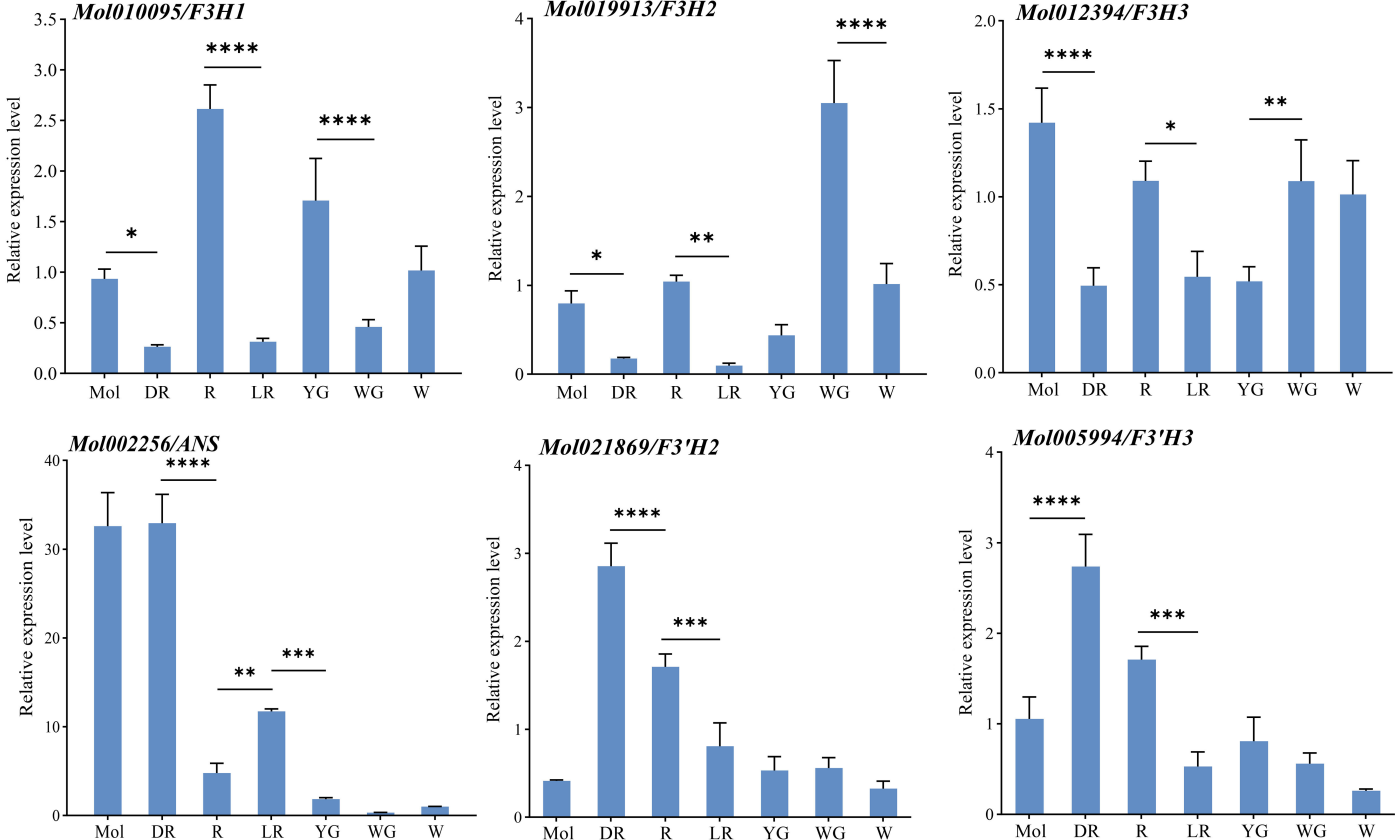
qRCR analysis of the expression of six structural genes in tepals of *C. haematodes* and *C. sinense*. * indicates *P* < 0.05, ** indicates *P* < 0.01, *** indicates *P* < 0.001, and **** indicates *P* < 0.0001. Mol, *C. sinense* ‘Qihei’; deep red (DR), red (R), light red (LR), yellow-green (YG), and white-green (WG), *C. haematodes*; and white (W) refer to accessions of *C. tortisepalum*.

To further verify the expression of structural genes in *C. sinense* and *C. haematodes* with different flower colors (DR, R, LR, YG, WG and W), qRT-PCR analysis was performed (**Figure 7**). The results showed that there were significant differences in the expression levels of each structural gene in tepals with different colors. Compared with *C. sinense* and the light colors of *C. haematodes*, *CsF3’H2* genes showed significantly higher expression levels in both DR and R. Nonetheless, *CsF3’H2* was lower expressed than *CsF3H1* in *C. sinense*, indicating a more specific role in the flower color regulation in *C. haematodes*. The expression levels of *CsF3H* genes in *C. sinense* and R was higher than that of DR. Among them, the expression level of *CsF3H1* was the highest in R, while the expression level of *CsF3H2* was significantly higher in white WG. These results were largely consistent with transcriptome sequencing data. Nonetheless, the expression of *CsANS* (*Mol002256*) in *C. sinense* and DR were quite similar, both were significantly higher than that in other colors of *C. haematodes*. Thereby, *CsANS* may also play an important role in the determination of dark red flower colors in *C. sinense* and *C. haematodes*.

### 2.7 Subcellular localization of structure gene-encoded proteins

**Figure 8:**
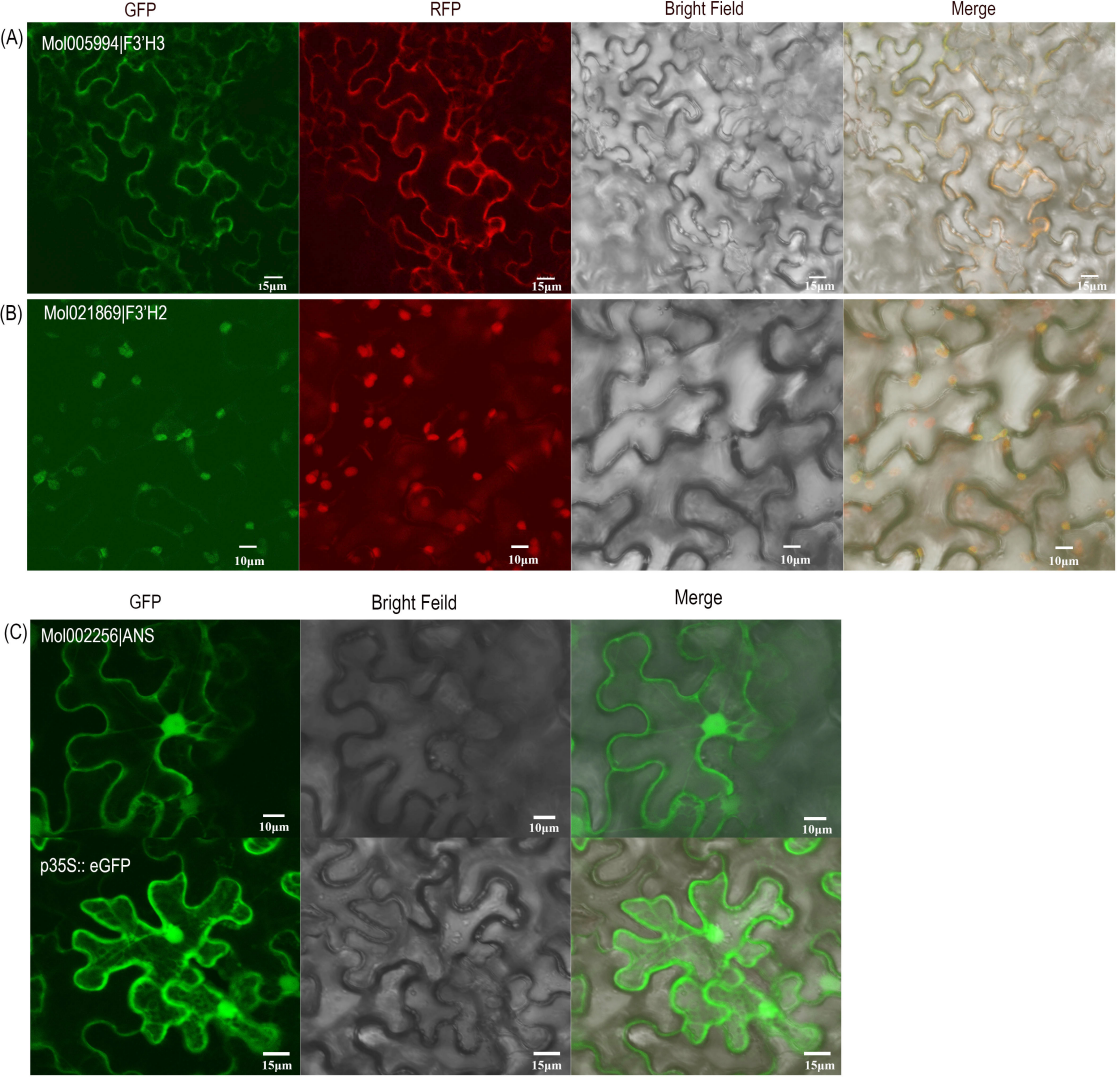
Subcellular localization of *C*. *haematodes* 2-ODD proteins. Gene-GFP fusion constructs were transiently expressed in the epidermis cells of *Nicotiana benthamiana*. **(A, B)** Localization of CsF3’H3 **(A)** and CsF3’H2 **(B)** fusion protein. From left to right: green fluorescence of gene-GFP fusion constructs, red fluorescence of the cell membrane marker DsRed-RFP **(A)** and chloroplast marker-RFP **(B)**, bright-field, and merged microscope images. **(C)** Localization of CsANS fusion protein. The 35S::GFP vector was used as control.

Three different structure gene-encoded proteins (CsANS, CsF3’H2, and CsF3’H3) were further selected for subcellular localization analysis. All the three cloned proteins were transient expressed in the epidermal cells of *Nicotiana benthamiana* leaves by using a 35S::GFP vector. The results revealed that CsF3’H3 is a cell membrane protein (**Figure 8A**), suggesting its potentially function involved in cell signaling process or other membrane-associated processes. On the other hand, CsF3’H2 is localized to the chloroplast (**Figure 8B**), which may have participated in plastid-related processes. Meanwhile, the diffuse CsANS-GFP fluorescence distributed throughout the cytoplasm, without overlapping with organelle-specific markers (**Figure 8C**). This suggests that ANS functions as a soluble cytoplasmic protein or may transiently interact with other cellular components under specific conditions. These findings provide crucial insights for further functional characterization of the structural genes in the tepals of *C. haematodes*.

## 3 Discussion

As secondary metabolites of plants, pigments are highly diverse in nature, but plant coloration is primarily governed by three major classes of pigments: carotenoids, betalains, and flavonoids (Tanaka et al., 2008). Plant organs such as roots, stems, and leaves generally contain substantial amounts of carotenoids and flavonoids. In contrast, flower coloration in many plant species largely depends on the content of flavonoids. Anthocyanins serve as the primary chromogenic compounds in flavonoids, imparting a wide range of colors to plant tissues through the C6-C3-C6 tri-cyclic structure (Tanaka et al., 2008). In this study, we investigated the pigment contents, including anthocyanins, carotenoids and chlorophylls, in various flower colors of *C. haematodes* and *C. sinense*. The results highlighted the pivotal role of anthocyanins in the red coloration in *C. haematodes*, with significantly higher concentrations observed in dark red (DR) and red (R) tepals compared to lighter or non-red variants (**Figure 1**). This study enhances our understanding of how these pigments contribute to flower color diversity within the genus *Cymbidium*.

The biosynthesis pathway of plant anthocyanins has been extensively studied, and the metabolites of each branch are catalyzed by a series of enzymes encoded by structural genes. Recent investigations into *Cymbidium* have identified key structural genes (e.g., *CsFLS*, *CsANS*, *CsF3H*) and regulatory *MYBs* (e.g., *CgMYB91*, *CeMYB22*) associated with anthocyanin biosynthesis (Chen et al., 2022; Zheng et al., 2023). In this study, 20 structural genes related to anthocyanin biosynthesis in *C. haematodes* were identified from transcriptome sequencing data (**Table 1**), the number of which was similar to that of *Dendrobium officinale* (Yu et al., 2018; Yu et al., 2021), *Phalaenopsis* (Chao et al., 2018) and other orchids. These genes including *CsCHS*, *CsF3’H*, *CsDFR*, and *CsANS* exhibited distinct expression patterns and sub-functionalization. Phylogenetic analysis revealed that the structural genes of *C. haematodes* share high homology with those of closely related *Cymbidium* species, suggesting conserved evolutionary functions (**Figures 3** and **4**). For instance, *CsCHS1* was clustered closely with *CyCHS* from *Cymbidium hybrid*, while *CsUFGT4* showed high homology with that in *Dendrobium* species. This indicates there exist both evolutionary conservation and potential functional diversification within the Orchidaceae family. Notably, the 2-ODDs, including CsF3H, CsANS, and CsFLS, contained conserved residues (H-x-D-xn-H and R-x-S) essential for enzyme activity, further supporting their roles in flavonoid metabolism.

Previous studies revealed that structural genes such as *CHS*, *F3’H*, *DFR*, and *UFGT* promoted anthocyanin biosynthesis in orchid species like *Oncidium*, *Cymbidium* and *Phalaenopsis*, which was consistent with the flower color phenotypes (Ma et al., 2009; Chen et al., 2011; Liu et al., 2012). For example, up-regulation of *CHS* correlates with red pigmentation in the flowers of *C. ensifolium* (Li et al., 2018) and *Oncidium* (Li et al., 2012), while differential expression of *F3H* explained color intensity variations in *Vanda* (Junka et al., 2011) and albino phenotypes in *Paphiopedilum* (Li et al., 2021). In this study, we found that *Mol010095* (*CsF3H*) showed a significantly higher expression level in R and *C. sinense*, whereas very low expression levels in WG. It was consistent with the finding that the expression level of *Va-F3H1* in *Vanda* was higher in light purple-red varieties than that in white varieties (Junka et al., 2011). F3’H determines anthocyanin hydroxylation patterns, thereby affecting outcome colors. In *Phalaenopsis*, *PhF3’H* expression is markedly higher in red cultivars than in yellow ones, resulting in facilitation of cyanidin and peonidin biosynthesis in red ones (Yang et al., 2013). DFR catalyzes the conversion of colorless leucoanthocyanidins to colored anthocyanidins. Furthermore, ANS is indispensable for final anthocyanidin formation. For instance, high *ANS* expression in *Cymbidium* ‘Fuyun Danxia’ correlates with purple-red flowers (Kong et al., 2021). Transient expression of *CHS*, *DFR*, as well as *ANS* in *C. kanran* induced reddish-purple pigmentation in white tissues (Zhou et al., 2021). UDP-glycosyltransferase (UFGT) stabilizes anthocyanins via glycosylation. Silencing *PeUFGT3* in *Phalaenopsis* reduced anthocyanin content, leading to faded red petals (Chen et al., 2011). In this study, expression profiles revealed that *CsF3’H2* and *CsF3’H3* were highly expressed in red tepals of *C. haematodes*, suggesting their pivotal roles in anthocyanin accumulation. Nonetheless, proteins encoding by these two genes have different subcellular localizations, with CsF3’H2 localized to chloroplasts, and CsF3’H3 localized to the cell membrane (**Figure 8**). It suggests these two proteins may have diverse roles in anthocyanin biosynthesis and transport. In contrast, the LBG genes such as *CsDFR* and *CsUFGTs* were more highly expressed in *C. sinense*, indicating the presence of species-specific regulatory mechanisms. The results from qPCR results further supported these observations, revealing that *CsF3’H* genes displayed highly expression in DR and R tepals. Notably, *CsANS* expression peaked in DR and *C. sinense*, indicating its critical role in the final formation of anthocyanins, as previously demonstrated in *C. kanran* (Zhou et al., 2021) and *Cymbidium* ‘Fuyun Danxia’ (Kong et al., 2021). Therefore, it is speculated that the two *CsF3’H* and *CsANS* genes may serve as key structural genes for anthocyanin glycoside metabolism in *C. haematodes*.

## 4 Materials and methods

### 4.1 Plant materials

In this study, the floral tepals of different *Cymbidium* species with various colors including *C. haematodes*, *C. kanran*, *C. tortisepalum*, and *C. sinense* were used as the experimental materials listed in **Table 4**. These *Cymbidium* species include five native *C. haematodes* accessions, two *C. kanran* accessions and two *C. tortisepalum* accessions with different flower colors and one *C. sinense* variety ‘Qihei’ (blackish red). The *C. sinense* variety ‘Qihei’ is native to Fujian province (China), whereas the *C. haematodes* accessions that collected from Yunnan province (China) have not been officially named yet, so they are numbered and represented by different flower colors: dark red (DR), red (R), light red (LR), yellow-green (YG), white-green (WG), and white (W). Likewise, the *C. kanran* and *C. tortisepalum* accessions were also presented by flower colors as *C. kanran* DR (dark red), *C. kanran* G (green), *C. tortisepalum* W (white) and *C. tortisepalum* R (red). The plant materials used in this study were all cultivated in green houses at the Orchid Germplasm Resources Paddy of Zhejiang Province (Hangzhou, China). Healthy and disease-free individuals from different flower color accessions of *C. haematodes* were selected. Due to the different flowering periods of *Cymbidium* species, the tepals were all sampled 10 days after flowering. Samples are taken from the perianth lobes on the same blooming condition, including sepals and petals. Three biological replicates were taken for each flower color. The samples of different flower color series of *Cymbidium* species were collected. The samples were quickly placed in liquid nitrogen for drying and freezing, and then stored at -80°C refrigerator for further analysis.

**Table 4:** Information of the *Cymbidium* species used in the present study.

Number	Sample name	Species	Variety	Origin
1	*C. haematodes*-DR	*C. haematodes*	native species	Yunnan, China
2	*C. haematodes*-R		native species	Yunnan, China
3	*C. haematodes*-LR		native species	Yunnan, China
4	*C. haematodes*-YG		native species	Yunnan, China
5	*C. haematodes*-WG		native species	Yunnan, China
6	*C.kanran*-DR	*C.kanran*	large-leaved variety	Zhejiang, China
7	*C.kanran*-G		small-leaved variety	Zhejiang, China
8	*C.sinense*-Mol	*C.sinense*	qihei	Fujian, China
9	*C.tortisepalum*-W	*C.tortisepalum*	da xuesu	Sichuan, China
10	*C.tortisepalum*-R		native species	Sichuan, China

### 4.2 Determination of pigment types and contents

Each of 0.2 g samples of the tepals from different *C. haematodes* accessions (DR, R, LR, YG, and WG), *C. kanran* accessions (DR and G), *C. tortisepalum* accessions (R and W), and *C. sinense* “Qihei” were weighed, respectively. The samples were ground into powder quickly, with liquid nitrogen. Then, the anthocyanin contents in each sample were determined by colorimetry following the method described by Chen et al (Chen et al., 2016), and the total contents of chlorophyll and carotenoids were determined by spectrophotometry using modified protocols according to Wang (2006). Finally, the contents of main pigments in *Cymbidium* orchids were measured. The data were further analyzed using Microsoft Excel 2019 (Microsoft Corp., Redmond, WA) and GraphPad Prism software (vs. 8.4.3).

### 4.3 Identification of key structural genes in anthocyanin biosynthesis

Based on the quantitative analysis of anthocyanin contents in *C. haematodes*, the structural genes in the anthocyanin biosynthesis pathway were obtained by searching for the gene annotation in the NR database of *C. haematodes* transcriptome data. The corresponding nucleotide sequences were obtained from *C. sinense* genome annotation file using TBtools software (Wang, 2006), and the conserved domains in amino acid sequences of these genes were predicted using NCBI CDD (https://www.ncbi.nlm.nih.gov/Structure/cdd/wrpsb.cgi). The basic physicochemical properties of the identified protein sequences, such as molecular weight (MW) and isoelectric point (pI), were analyzed using ExPASy proteomics website (https://web.expasy.org/). Subcellular localization was predicted using online website Cell-PLoc2.0 (http://www.csbio.sjtu.edu.cn/bioinf/Cell-PLoc-2/).

### 4.4 Phylogenetic analysis of the structural genes

The amino acid sequences of reported structural genes from other plants including monocots and dicots were obtained from TAIR (http://www.arabidopsis.org/) and NCBI (https://www.ncbi.nlm.nih.gov/). CHS proteins from other species include *Arabidopsis thaliana* (AtCHS), *Petunia hybrida*, (PhCHS), *Vitis vinife*ra (VvCHS), *Antirrhinum majus* (AmCHS), *Oncidium* var. Gower Ramsey (OgCHS), *Paphiopedilum concolor* (PcCHS), *Dendrobium catenatum* (DcCHS), *Cymbidium hybrid* (CyCHS), and *Phalaenopsis equestris* (PeCHS). CHI proteins from other species include AtCHI (*A. thaliana*), PhCHI (*P. hybrida*), DcCHI (*D. catenatum*), PeCHI (*P. equestris*), AsCHI (*Apostasia shenzhenica*), CyCHI (*C. hybrid*), and DhCHI (*D. hybrid*). F3H proteins from other species include AtF3H (*A. thaliana*), PhF3H (*P. hybrida*), VvF3H (*V. vinife*ra), CyF3H (*C. hybrid*), DhF3H (*D. hybrid*), DoF3H (*D. officinale*), and AsF3H (*A. shenzhenica*). F3’H proteins from other species include AtF3’H (*A. thaliana*), PhF3’H (*P. hybrida*), VvF3’H (*V. vinife*ra), LsF3’H (*Lilium* sp*eciosum*), CyF3’H (*C. hybrid*), DhF3H (*D. hybrid*), DcF3’H (*D. catenatum*), and AsF3’H (*A. shenzhenica*). DFR proteins from other species include AtDFR (*A. thaliana*), PhDFR (*P. hybrida*), AmDFR (*A. majus*), OhDFR (*O.hybrid*), CyDFR (*C. hybrid*), DcDFRA (*D. catenatum*), AsDFR (*A. shenzhenica*), and PeDFR (*P. equestris*). ANS proteins from other species include AtANS (*A. thaliana*), PhANS (*P. hybrida*), LsANS (*L.* sp*eciosum*), PeANS (*P. equestris*), CyANS (*C. hybrid*), DoANS (*D. officinale*), OhANS (*O.hybrid*), and DcANS (*D. catenatum*). UFGT proteins from other species include AtUG3GT (*A. thaliana*), NnUFGT (*Nelumbo nucifera*), VvUFGT (*V. vinife*ra), and DcUFGT (*D. catenatum*).

The phylogenetic tree for plant *2-ODD* gene family members was constructed with protein sequences from 43 species including four *Cymbidium* species (*C. haematodes*, *C. sinense*, *C. ensifolium*, and *C. goeringri*), and *D. officinale* (Do), *P. equestris* (Pe), *A. shenzhenica* (As), *Sorghum bicolor* (Sb), *Setaria italica* (Si), *Ginkgo biloba* (Gb), *N. nucifera* (Nn), *Allium cepa* (Ac), *A. thaliana* (At), *Oryza sativa* (Os), *P. x hybrida* (Ph), *A. majus* (Am), *Zea mays* (Zm), *Rosa chinensis* (Rc), *V. vinifera* (Vv), *Fragaria x ananassa* (Fa), *Prunus cerasifera* (Pc), *Eustoma grandiflorum* (Eg), *Solanum lycopersicum* (Sl), *Camellia nitidissima* (Cn), *Gossypium hirsutum* (Gh), *Daucus carota* (Dc), *Vaccinium corymbosum* (Vc), *Paeonia lactiflora* (Pl), *Arachis hypogaea* (Ah), *Hibiscus sabdariffa* (Hs), *Salvia* sp*lendens* (Ss), *Lagerstroemia indica* (Li), *Ipomoea batatas* (Ib), *Strelitzia reginae* (Sr), *Brassica napus* (Bna), *Lilium regale* (Lr), *Glycine max* (Gm), *Punica granatum* (Pg), *Lycium chinense* (Lc), *Solanum tuberosum* (St), *Citrus sinensis* (Cs), and *Malus domestica* (Md), by using a MEGAX software (V10.0, Tokyo Metropolitan University, Tokyo, Japan). MUSCLE program was used for multiple sequence alignment, and the Maximum Likelihood (ML) method was used with 1000 bootstrap replicates. The phylogenetic trees were visualized and modified using MEGAX and iTOL (https://itol.embl.de/index.shtml).

### 4.5 Analysis of gene structure and conserved motifs of the 2-ODDs

To obtain the conserved amino acid sequences of the DNA-binding domain, the ClustalX program was used to conduct multiple sequence alignment of the 2-ODD (ANS, F3H, and FLS) protein sequences. The sequences of the related structural genes of the homologous species *C. goeringii* and *C. ensifolium* were obtained using a TBtools software (Chen et al., 2023). The conserved motifs of the homologous sequences of genes in *C. haematodes*, *C. goeringii* and *C. ensifolium* were analyzed through MEME online website (http://meme-suite.org/tools/meme). The maximum value of the motifs was set to 10, and the rest were default parameters. The results were visualized by TBtools software (Chen et al., 2023).

### 4.6 Expression analysis

Transcriptome sequencing was performed by using the tepals of *C. sinense* ‘Qihei’ and five different *C. haematodes* accessions (DR, R, LR, YG, and WG). The genome of *C. sinense* was used as the reference genome. Three biological replicates were performed for each sample. The total RNA of 18 samples was extracted according to Wang et al. (2022) and sequenced by Mateware Metabolic Biotechnology Co., LTD (Wuhan, China). Expression profiles of the 20 structural genes were obtained by FPKM (Fragments Per Kilobase of exon per million fragments Mapped) values and the Cufflinks software (http://cole-trapnell-lab.github.io/cufflinks, v2.2.1). The expression heatmap of structural genes was constructed using the TBtools software based on the FPKM values.

### 4.7 cDNA synthesis and qRT-PCR

A total of 21 samples from plant materials with seven different flower colors, namely *C. sinense* (Mol), DR, R, LR, YG, WG, and W, were selected for qRT-PCR analysis of candidate structural genes in anthocyanin biosynthesis. There were three biological replicates for each flower color accession. Total RNA was extracted from each sample using the RNA extraction kit according to Wang et al (Wang et al., 2022), and the extracted RNA was subjected to electrophoresis for quality detection. The RNA with qualified quality was reverse transcribed into cDNA using a cDNA synthesis system (Invitrogen, Shanghai, China). Primer Premier 5 software was used to design qRT-PCR primers for the selected structural genes. The primers were synthesized and sequenced by Genepioneer Biotechnologies Co., Ltd (Nanjing, China) (**Table 5**). The 20 μL reaction system for qRT-PCR amplification was as follows: 1 μL template, 1 μL each of upstream and downstream primers, 10 μL of 2×SYBR Premix Ex Taq fluorescent dye, and 7 μL ddH_2_O. The PCR amplification conditions were set by two-step method: 5 min of pre-denaturation at 95 °C, followed by 40 cycles of 10 s at 95 °C, 30 s at 60 °C, and 15 s at 95 °C, and then a melting curve program of 95 °C 15 s, 60 °C 60 s, and 95 °C 15 s. *Mol017122* gene was chosen as the internal standard gene to analyze the relative fold differences. The reaction system and amplification program were the same as above. Each sample was repeated three times. The relative expression levels of the target genes were calculated using the 2^-ΔΔCt^ method, and the data were analyzed and plotted using the Graphpad Prism software (vs. 8.4.3).

**Table 5:** Primer sequences for qRT-PCR.

Primer name	Primer sequences (5’-3’)	PCR Product size (bp)
Mol017122-F	AAGCCGTTCTGTCCCTTTATG	212
Mol017122-R	TTCTCGCTCTGCGGTTGTT	
Mol002256/ANS-F	GGGGCAGATTCAGGGCTAT	194
Mol002256/ANS-R	ACATTTTTGAGGCGAGGGTT	
Mol012394/F3H3-F	CGAGTTCAGCGACGAGATTC	108
Mol012394/F3H3-R	TCACACGCCTCCACGATTC	
Mol019913/F3H2-F	TCTTCACTTACAGCCCCGC	114
Mol019913/F3H2-R	TGACCCTCGACCGTTCATC	
Mol010095/F3H1-F	CGTCAATCCCCAACCCAA	128
Mol010095/F3H1-R	CCACTGACATTCTCACCCTCTC	
Mol021869/F3*’*H2-F	CCAGGATCTCATCTTCGCCC	136
Mol021869/F3*’*H2-R	CCGCACAAATCTACCCACCT	
Mol005994/F3*’*H3-F	AGAGTGCTGTCGAAGATGGC	125
Mol005994/F3*’*H3-R	AGCCTTGATGTCAGTGTCCG	

### 4.8 Subcellular localization

Three key structural gene-encoded proteins (CsANS, CsF3’H2, and CsF3’H3) were selected for subcellular localization analysis. An artificial green fluorescent protein (GFP) was fused in-frame to the 3’-terminus of each target gene. The pCAMBIA1300-eGFP plasmid was digested with the BamHI restriction enzyme (TAKARA FastDigest™) in a single-enzyme reaction. Gene-specific homologous recombination primers were designed based on the target gene sequences using CE Design software. The target genes were subsequently amplified and cloned into the linearized pCAMBIA1300-eGFP vector via homologous recombination. The 35S promoter was employed to drive the expression of the three genes. The DsRed-RFP vector was used as nucleus marker. To validate subcellular localization, co-localization experiments were performed using either a nuclear/tonoplast/chloroplast specific marker or free GFP as a cytoplasmic reference control. The constructed vectors were transiently expressed in the epidermal cells of *Nicotiana benthamiana* through *Agrobacterium*-mediated transformation. Fluorescence of fusion protein constructs and the makers were detected under Olympus confocal laser microscope FV3000 (Hachioji City, Tokyo, Japan).

### 4.9 Statistical analysis

Statistics were performed with Graphpad Prism software (vs. 8.4.3) using one-way ANOVA (*P* < 0.05). Significant differences in values were indicated by * (*P* < 0.05), ** (*P* < 0.01), *** (*P* < 0.001), and **** (*P* < 0.0001). Expression analysis was performed using mean ± standard deviation (SD) values of three biological replicates.

## 5 Conclusions

In conclusion, this study systematically identified and characterized key structural genes in the ABP of *C. haematodes*, revealing their functional divergence and evolutionary conservation. The differential expression patterns and subcellular localization of these genes highlight their specialized roles in flower color formation. Future research should focus on functional validation, such as gene knockout or over-expression of these key structural genes, to further elucidate their roles in regulatory networks and explore potential applications in orchid breeding aimed at developing novel flower colors.

## Data Availability

The datasets presented in this study can be found in online repositories. The names of the repository/repositories and accession number(s) can be found in the article/supplementary material.
